# Cytotoxic T-lymphocyte elicited vaccine against SARS-CoV-2 employing immunoinformatics framework

**DOI:** 10.1038/s41598-021-86986-6

**Published:** 2021-04-07

**Authors:** Neeraj Kumar, Nikita Admane, Anchala Kumari, Damini Sood, Sonam Grover, Vijay Kumar Prajapati, Ramesh Chandra, Abhinav Grover

**Affiliations:** 1 Department of Chemistry, University of Delhi, Delhi, 110007 India; 2 Department of Biotechnology, Jawahar Lal Nehru University, Delhi, 110067 India; 3 JH-Institute of Molecular Medicine, Jamia Hamdard, Hamdard Nagar, Delhi, 110062 India; 4 Department of Biochemistry, School of Life Sciences, Central University of Rajasthan, Ajmer, 305817 Rajasthan India

**Keywords:** Computational biology and bioinformatics, Biophysics

## Abstract

Development of effective counteragents against the novel coronavirus disease (COVID-19) caused by severe acute respiratory syndrome coronavirus 2 (SARS-CoV-2) strains, requires clear insights and information for understanding the immune responses associated with it. This global pandemic has pushed the healthcare system and restricted the movement of people and succumbing of the available therapeutics utterly warrants the development of a potential vaccine to contest the deadly situation. In the present study, highly efficacious, immunodominant cytotoxic T-lymphocyte (CTL) epitopes were predicted by advanced immunoinformatics assays using the spike glycoprotein of SARS-CoV2, generating a robust and specific immune response with convincing immunological parameters (Antigenicity, TAP affinity, MHC binder) engendering an efficient viral vaccine. The molecular docking studies show strong binding of the CTL construct with MHC-1 and host membrane specific TLR2 receptors. The molecular dynamics simulation in an explicit system confirmed the stable and robust binding of CTL epitope with TLR2. Steep magnitude RMSD variation and compelling residual fluctuations existed in terminal residues and various loops of the β linker segments of TLR2-epitope (residues 105-156 and 239-254) to about 0.4 nm. The reduced R_g_ value (3.3 nm) and stagnant SASA analysis (275 nm/S^2^/N after 8 ns and 5 ns) for protein surface and its orientation in the exposed and buried regions suggests more compactness due to the strong binding interaction of the epitope. The CTL vaccine candidate establishes a high capability to elicit the critical immune regulators, like T-cells and memory cells as proven by the in silico immunization assays and can be further corroborated through in vitro and in vivo assays.

## Introduction

The outbreak of 2019 novel coronavirus (SARS-CoV2; Family Coronaviridae) in Wuhan, China, has raised intense attention and global emergency pertaining to its highly infectious nature^1,2^. A similar outbreak was seen in 2003 when coronavirus affected people with the severe acute respiratory syndrome (SARS)^3^. This contagious virus after its third introduction into the human society has outrageously caused a global pandemic severely affecting the health care systems and global economy. The virus is instigating a severe respiratory ailment where the infected, asymptomatic individuals are also capable of viral transmission^4^. As per the latest statistics by 18 October, global cases have risen up to 40 million with 1.1 million deceased and 2.4 million new reports, suggesting vigorous status of the infectious virus (World Health Organisation, WHO 2020) which has favoured its transmission to 188 countries and 25 territories around the globe^5^. As a characteristic of coronaviruses, SARS-CoV2 also possesses a surface spike glycoprotein responsible for the pathogenesis by mediating the binding of it to host cellular receptors leading to its fusion with the host cell membrane. The SARS-CoV spike (S) glycoprotein majorly comprises of two subunits; the S1 subunit acts as a receptor-binding domain that participates in the binding to SARS-CoV2 harboring angiotensin-converting enzyme 2 (ACE2) receptors of the host, and the S2 subunit facilitates the fusion of viral and host cell membranes^6,7^. During the SARS-CoV infection, the S glycoprotein arbitrates neutralizing-antibody and T-cell responses evolving protective immunity. The S glycoprotein possesses several conformational epitopes critical for the recognition as well as abrogation of viral infection, which makes it a potential target for designing antigenic determinants and engineer them into effective subunit vaccines^8,9^.

It is noteworthy that the mechanistic understanding of this viral infection has taken an enormous leap after two precedents. Still, the present scenario has created an emergency and posed tremendous challenges for developing effective prevention, treatment, and epidemiological control^4^. The scientific community has rigorously developed various approaches like drug repurposing for treating and managing the deadly viral infection, but the most critical step is to develop effective vaccines that would abrogate its recurrent infections. In the past few decades, rigorous efforts for developing vaccines against the infectious coronaviruses have been explicitly made for targeting MERS and SARS, but no licensed antiviral treatment or specific vaccine could be developed yet for the effective management of COVID-19^10,11^. This swift epidemical outbreak is taking an enormous toll on the scientific community, and a key tailback for effective vaccine development is the dearth of access to samples from infected patients. In such a situation, in silico estimation of vaccine targets can be highly advantageous and serve as a guidance to the scientific community for designing a robust, timely, rapid and cost-effective solution in the scarcity of resources^12^.

The recent strategies of treatment also involve the convalescent plasma therapy where the plasma-derived from recently recovered patients is introduced into other infected patients for delivering the anti-corona antibodies^13,14^. Similarly, this study is based on designing vaccine constructs, precisely characteristic to the immune signatures or antigenic determinants of the SARS-CoV2, which could be directly administered into the host for generating a highly specific immune response capable of immunizing the host against a subsequent viral infection.

Such epitope-driven, immunome-based vaccines can open new avenues for targeting the infectious or rapidly mutating pathogens, particularly by targeting their conserved epitopes and providing highly specific but competent immune responses^15–18^. Furthermore, to authenticate this host cell specificity and binding efficiency of the vaccine constructs with the target receptors of host cell surfaces, we performed molecular interaction studies with MHC-I and MHC-II receptors^19,20^. Also, to validate our results, we performed the binding analysis of the vaccine with the fundamental sensor molecules of the host, Toll-like receptor2 (TLR2). TLR-2 is a membrane-bound receptor that is implicated in the early interplay of host responses deciphering innate or adaptive immunity by identifying the viral antigens and affecting viral pathogenesis. TLR-2 binding with the ligand triggers the regulatory network and stimulates the production of interferons IFN- β and interleukins to destruct viral pathogens^21,22^. Recent studies have delineated the use of in silico approaches in designing potent multi-epitope vaccines targeting SARS-CoV-2, putatively generating strong immune responses^23,24^.

Several studies have reported a preferential impact on CD8^+^ T cells or CTL specific lymphopenia and a reduced cytotoxic potential was noted predominantly in patients with COVID-19 who required intensive care^25,26^. Bridging the former evidences demanding specific CD8^+^ T cell responses in SARS-CoV-2 infections, the present study focuses primarily on the top-scoring CTL epitopes predicted using five different algorithms and subsequent identification of highly efficient common consensus epitopes. The use of molecular docking and molecular dynamics simulation assays assist in evaluating the potency and specificity of vaccine candidate with target receptors^27^. The designed vaccine construct was assessed for specific and robust binding with MHC receptors (MHC-1 and MHC-2) and virus-specific membrane receptor TLR-2^28^. By using the computational advancement avenues, we also performed the in silico immunization assay for designing vaccine and assessed the strength of vaccine construct for eliciting the critical regulators of the immune system. Out of these significant results, the vaccine construct was well-characterized by physicochemical parameters. This study provides the breakthrough highly potential CTL vaccine against the COVID-19 by theoretical approaches.

## Results

The present study identified the potential cytotoxic T-lymphocyte immune signature from the infectious sequence of the SARS-CoV-2 strain, which could be used as antiviral therapeutics to evoke the specific immune response to fight against the coronavirus. Putative CTLs have been designed with advanced immunoinformatics approaches to be used as a vaccine candidate.

### Coronavirus infectious sequence retrieval and assessment

The infectious sequence of coronavirus was availed from the reports and molecular evidence of coronavirus from the protein database available on NCBI (National Centre for Biotechnology Information). The updated version of the infectious sequence of spike glycoprotein of coronavirus disease-2019 (COVID-19) from Wuhan, China, was retrieved with GenBank ID QHR63290.2. The spike glycoprotein of coronavirus is reported to play a vital role in infection and progression of virus to the host. It was said to isolate from the early-stage corona infected patients. Furthermore, we have analyzed the retrieved infectious sequence through the protein–protein BLAST analysis. The outcome results showed the spike glycoprotein sequence is substantially conserved and has high similarity with surface glycoprotein of severe acute respiratory syndrome coronavirus (> 100 infectious sequences) and Bat coronavirus genome. Moreover, pairwise sequence alignment through the different methods; neighbour-joining, tree evaluation and fast minimum evolution methods, we found it has high sequence similarity with Wuhan seafood market pneumonia virus for more than 172 hits and minimal variation of 0.0004–0.0005. These shreds of evidence signified the spike glycoprotein infectious sequence, a potential candidate for cytotoxic T-lymphocytes epitopes derivation, to evoke immune response against the coronavirus.

### Putative cytotoxic T-lymphocytes epitopes prediction

The Cytotoxic T-lymphocytes (CTL) epitopes can be used as a potential vaccine candidate to induce active immunity to fight against the coronavirus. Further, they provide the immunological memory to prepare the immune system against viral re-infection. To identify the most precise and specific CTLs, five different immunological algorithms-based servers were employed. The outcome CTLs from the five servers (Rankpep, BIMAS, NetMHC 4.0, CTLPred, and NetCTL-1.2) are enlisted in Table 1. Among the resulting CTLs, top-scored ten epitopes were shortlisted; FVSGNCDVV, FGGFNFSQI, TLDSKTQSL, SIIAYTMSL, MFVFLVLLP, FVFLVLLPL, FRVQPTESI, NYNYLYRLF, LTDEMIAQY, and WTAGAAAYY. Shortlisted top-scored CTLs were further investigated for various immunological parameters (Antigenicity, immunogenicity, C-terminal proteasomal affinity, and transporter associated with antigen processing, TAP affinity) essential for an efficacious vaccine.

**Table 1:** Putative cytotoxic T-lymphocytes epitopes for the infectious sequence of coronavirus identified by employing the five different algorithms.

Epitope rank	Rankpep	BIMAS	NetMHC 4.0	CTLpred	NetCTL-1.2
1	FVSGNCDVV	TLDSKTQSL	MFVFLVLLP	FRVQPTESI	LTDEMIAQY
2	FGGFNFSQI	SIIAYTMSL	FVFLVLLPL	NYNYLYRLF	WTAGAAAYY
3	FQFCNDPFL	GLTVLPPLL	VFLVLLPLV	AQVKQIYKT	TSNQVAVLY
4	KVGGNYNYL	VLNDILSRL	FLVLLPLVS	YTNSFTRGV	CVADYSVLY
5	CVNFNFNGL	SALEPLVDL	LVLLPLVSS	TRFQTLLAL	KTSVDCTMY
6	FCGKGYHLM	ALNTLVKQL	VLLPLVSSQ	NENGTITDA	STECSNLLL
7	VVNQNAQAL	MFVFLVLLP	LLPLVSSQC	YQTSNFRVQ	GAEHVNNSY
8	VLYENQKLI	GLTVLPPLL	LPLVSSQCV	KCYGVSPTK	NIDGYFKIY
9	YHKNNKSWM	WTFGAGAAL	PLVSSQCVN	GKIADYNYK	YSSANNCTF
10	VSGNCDVVI	SLSSTASAL	LVSSQCVNL	KSTNLVKNK	WMESEFRVY

Top ten ranked predicted epitopes from each server are depicted here.

### Optimization of CTL epitopes with immunological parameters

Top scored CTL epitopes were investigated for MHC binding, Transporter associated with antigen processing (TAP) affinity, C-terminal cleavage efficiency, antigenicity, and non-toxic nature characterization. The MHC binding affinity, TAP transport capability, and peptide C-terminal cleavage efficiency were analyzed using the netCTLpan server. The obtained results depicted the higher cleavage efficiency for antigen processing with a weighted score for proteasomal C-terminal affinity with a cut-off value of 0.225; the epitopes with higher scores are susceptible to proteasomal C-terminal sympathy. It will help in antigen processing into the short peptides (8–10 amino acids) for antigen presentation to Major Histocompatibility Complex-1 (MHC-1) T-cells to generate an immune response. The CTL epitopes were also evaluated for TAP affinity, which demonstrated the high TAP affinity of epitopes with a cut-off value of 0.025, shown in the Table 2. Also, we have performed the conservancy analysis of epitopes and in results; we found epitopes are highly conserved for multiple surface glycoprotein strains of SARS coronavirus-2 (more than 200 hits). In addition, we have also assessed the world population coverage (geographical locations) for vaccine construct through the IEDB (immune epitope database) analysis resource. In results, we found the conserved nature of epitope and high population coverage of 43.68% of the world through the epitope conservancy of the vaccine construct. These outcomes signify the applicability and potency of designed vaccine construct for large geographical populations.

**Table 2:** Optimization of CTL epitopes with immunological parameters for MHC binding, transporter associated with antigen processing (TAP) affinity, C-terminal cleavage efficiency, combinatorial rank, antigenicity, and non-toxic nature characterization.

	Peptide	MHC	TAP	Cle	Comb	%Rank	Antigenicity	Nature
1	FVSGNCDVV	0.49600	0.17200	0.73303	0.66523	3.00	Non-antigenic	Non-Toxin
**2**	**FGGFNFSQI**	**0.16600**	**0.25400**	**0.65415**	**0.30683**	**16.00**	**Highly-antigenic (1.27)**	**Non-Toxin**
**3**	**TLDSKTQSL**	**0.48400**	**0.58000**	**0.97582**	**0.71806**	**2.00**	**Highly-antigenic (1.06)**	**Non-Toxin**
4	SIIAYTMSL	0.77400	1.27400	0.97302	1.02478	0.15	0.5234	Non-Toxin
5	MFVFLVLLP	0.05000	0.39800	0.13377	0.09005	50.00	0.4166	Non-Toxin
6	FVFLVLLPL	0.68300	1.04400	0.97043	0.92745	0.80	Highly-antigenic (0.86)	Non-Toxin
7	FRVQPTESI	0.04400	0.75000	0.93979	0.27420	32.00	Highly-antigenic (0.93)	Non-Toxin
8	NYNYLYRLF	0.02500	2.73800	0.43657	0.19168	50.00	Non-antigenic	Non-Toxin
9	LTDEMIAQY	0.08000	2.65200	0.97316	0.36526	9.00	Non-antigenic	Non-Toxin
10	WTAGAAAYY	0.09900	2.83000	0.94703	0.38283	9.00	Highly-antigenic (0.630)	Non-Toxin

Moreover, epitopes were further analyzed for MHC binder affinity and antigenicity profiles through the VaxiJen server. Obtained results showed the high antigenic characteristics of the CTL epitope and MHC binding affinity with a threshold value of 0.4. After that nature of epitopes was examined which, depicted the non-toxic nature of all shortlisted epitopes using the Toxinpred server enlisted in Table 2. These significant results demonstrated the high ranked epitopes (FGGFNFSQI, TLDSKTQSL, FVFLVLLPL, FRVQPTESI, and WTAGAAAYY) with high immunological parameters. The high-rank epitopes were further examined for their specific binding with HLA-A*0201 receptor and virus infection host membrane specific toll-like receptor-2 (TLR2).

### Binding analysis of selected epitopes with HLA-A*0201

**Figure 1:**
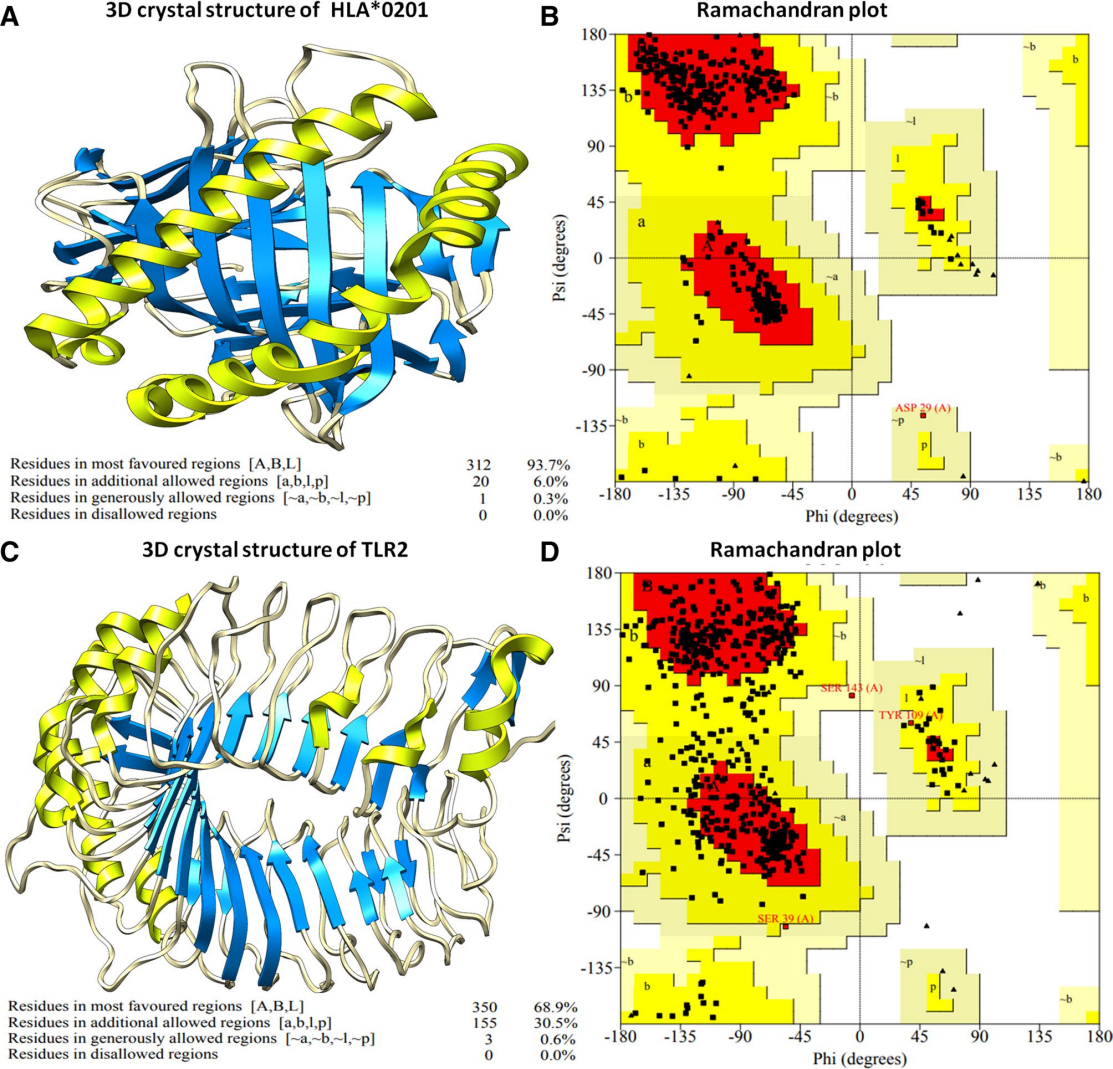
(**A**) Three-dimensional structure of HLA-A*0201 receptor. (**B**) Ramachandran plot analysis of HLA-A*0201 protein receptor, suggesting structure existence in the allowed region. (**C**) Three-dimensional structure of Toll-like receptor-2 receptor. (**D**) Ramachandran plot analysis of TLR2 receptor, presence in the permitted area with optimal quality of structures.

Selected CTL epitopes were investigated for binding with the HLA-A*0201 receptor to assess their specificity for humans. HLA-A*0201 is a major type of human major histocompatibility complex class-1 cell surface receptor. The three-dimensional structure of HLA-A*0201 was availed from the RCSB protein data bank with PDB ID 1I4F. Retrieved HLA-A*0201 structure available in co-crystal form was processed and prepared molecular docking analyses. HLA-A*0201 consisted of two chains; Chain-A of size 275 amino acids and Beta-2 microglobulin chain of size 100 amino acids. The processed HLA-A*0201 structure was analyzed for stereochemical parameters through the Ramachandran plot. The Ramachandran plot illustrated that 93.7% of residues of HLA-A*0201 lie in the favorable region, and 6.3% residues lie in the allowed region with no residues in the outlier region of the plot (Fig. 1).

Moreover, the Verfiy3D server also showed the high quality of structure with more than 97% residues having average 3D-1D scores ≥ 0.2. The optimized HLA-A*0201 structure was employed for binding analysis of selected epitopes using the HPEPDock server. The 3D structures of selected epitopes were modulated with the PepFold server. Designed 3D structures of CTL epitopes were energy minimized and optimized for molecular docking analysis with HLA-A*0201. HPEPDock server worked on the bases of the hierarchical algorithm for molecular docking with more than 72% success rate. The Top-scored high docking energy minimized models were analyzed for their molecular binding with the HLA-A*0201 receptor (Table 3). The obtained results depicted that CTL-1 and CTL-5 epitopes have the highest binding energy score of − 251.779 kJ/mol and − 246.834 kJ/mol, respectively. Among them, most proximal binding epitopes (CTL-1 and CTL-5) with the highest docking score were selected and further validated with virus infection specific host membrane receptor TLR-2 in humans.

**Table 3:** Molecular binding analyses of shortlisted CTL epitopes with HLA-A*0201 receptor.

S. No	Docked CTL models	CTL epitopes	Molecular docking scores with HLA-A*0201 receptor (kJ/mol)
1	CTL-1	FGGFNFSQI	− 251.779
2	CTL-2	TLDSKTQSL	− 172.727
3	CTL-3	FVFLVLLPL	− 240.909
4	CTL-4	FRVQPTESI	− 205.960
5	CTL-5	WTAGAAAYY	− 246.834

### Validation of lead epitopes with coronavirus infection specific human membrane TLR2 receptor

Toll-like receptors play a main regulatory role in viral proteins reception and further derivation of active immunity to diminish viruses. TLR2 interaction with the ligand evokes the critical regulators of the immune system and stimulates the interferons and interleukin to fight the infections. The lead epitopes were examined for their binding with the human TLR2 receptor. The 3D crystal stricter of TLR2 was retrieved from the protein data bank with PDB ID 1E3G and energy minimized and prepared for molecular docking assays. TLR2 structure was also assessed for the stereochemical properties. Ramachandran plot analysis depicted 68.9% residue of TLR2 structure lie in the favorable region; 31.1% residues lie in the allowed region and none residues in the outlier region. After that, lead epitopes CTL-1 and CTL5 were docked with the TLR2 receptor. Notably to strengthen our results and to define highly specific binder epitope, we employed three different molecular docking programs; HPEPDock, Hex 8.0, and Cluspro suite. HPEPDock worked based on the hierarchy algorithm, Hex 8.0 by fast Fourier transformation, and Cluspro worked based on piper rigid body methods. Outcome results depicted CTL-5 (WTAGAAAYY) interacted proximally to the binding groove of the TLR2 receptor from all three molecular docking methods.

**Figure 2:**
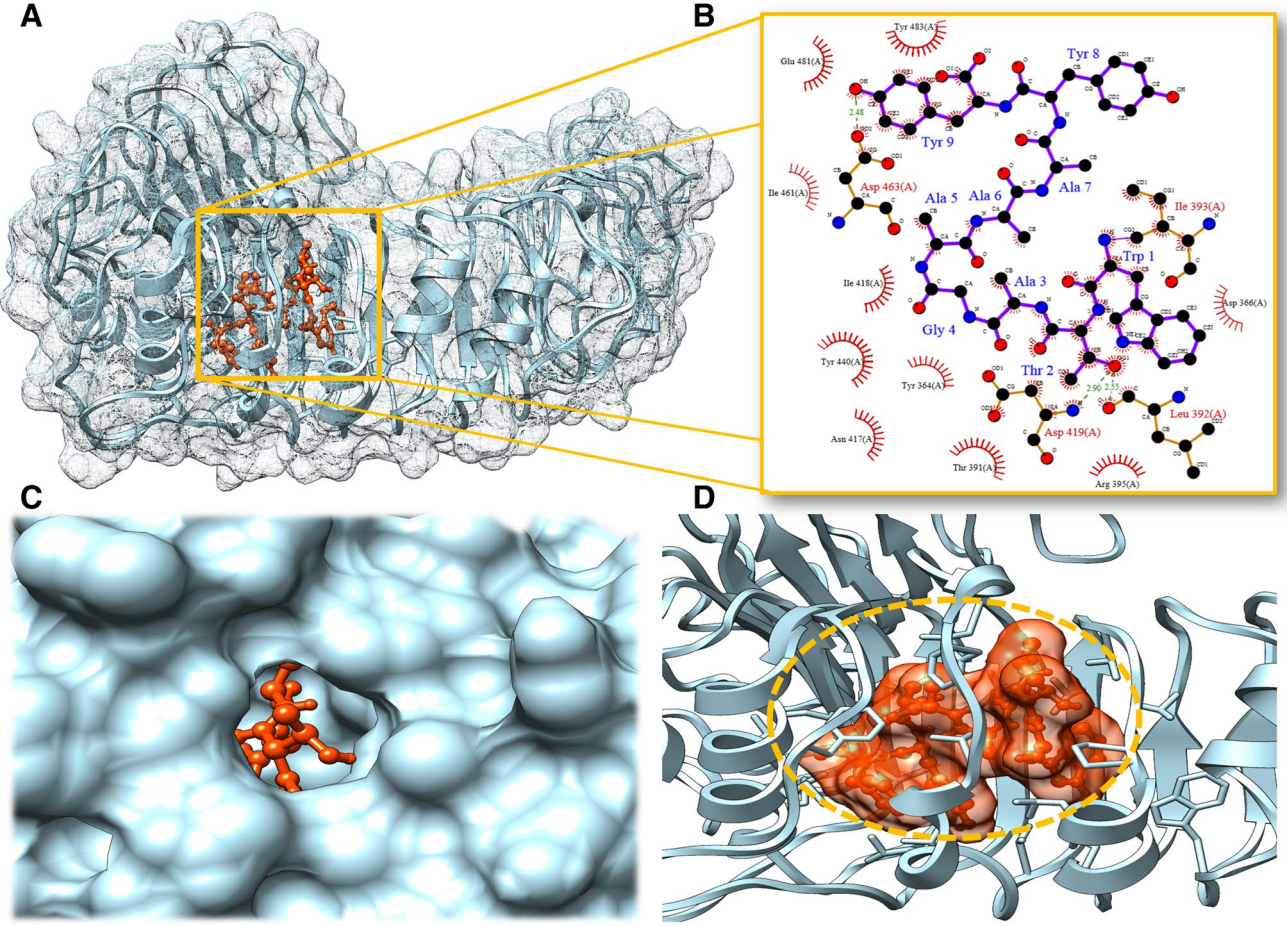
Binding analyses of lead CTL epitope with host membrane specific TLR2 receptor (**A**) Interacting complex of lead CTL epitope with TLR2, mesh network, TLR2 residues shown in sky blue and CTL epitope in orange color. (**B**) Molecular interactions involved in the strong binding of lead CTL epitope to TLR2 receptor. (**C**) Surface view of a binding grove of TLR2 during interaction of CTL epitope. (**D**) CTL epitope binding cavity network responsible strong and stable binding with TLR2.

Moreover, we analyzed and compared the top-scored CTL-1 and CTL-5 though Ligplot and Chimera molecular modeling suite. Obtained results showed the strong interaction of CLT-5 than CTL-1 with the involvement of large hydrogen bonds and hydrophobic interactions. CTL-5 formed the three hydrogen bonds with TLR2 receptor; first Thr2 with Leu392 residue of TLR2 of bond length 2.55 Å, Secoddn with Thr2 with Asp419 residue of TLR2 of bond length 2.90 Å and Tyr9 with Asp463 residue of TLR2 of bond length 2.48 Å. Also, CTL-5 showed the involvement of a large number of Hydrophobic interactions (Fig. 2). Besides, the CTL-1 epitope only showed the formation of two hydrogen bonds and lesser hydrophobic interactions shown in the Table 4. These outcomes significantly elucidated the potential CTL binder with host-specific membrane receptors.

**Table 4:** Molecular binding analyses of shortlisted lead CTL epitopes with human TLR2 receptor with different docking programs.

TLR2 receptor	CTL epitope	HPEPDock score (kJ/mol)	HEX 8.0 score (kJ/mol)	Cluspro score (kcal/mol)	Hydrogen bonds	Hydrophobic interaction
CTL model-1	FGGFNFSQI	− 187.772	− 311.25	− 848.6	Gly2-Thr416 of bond length 2.32 Å	Arg340, Gln363, Tyr364, Ile393, Ile461, Lys439, Tyr440 and Gln481
					Gly3-Gln390 of bond length 2.22 Å	
CTL model-5	WTAGAAAYY	− 210.148	− 360.37	− 911.5	Thr2-Leu392 of bond length 2.55 Å	Tyr364, Thr391, Leu392, Ile393, Arg395, Asn417, Ile418, Tyr440, Ile 461, Glu481 and Tyr483
					Thr2-Asp419 of bond length 2.90 Å	
					Tyr9-Asp463 of bond length 2.48 Å	

### Molecular dynamics simulation of TLR2 and TLR2-epitope complex

**Figure 3:**
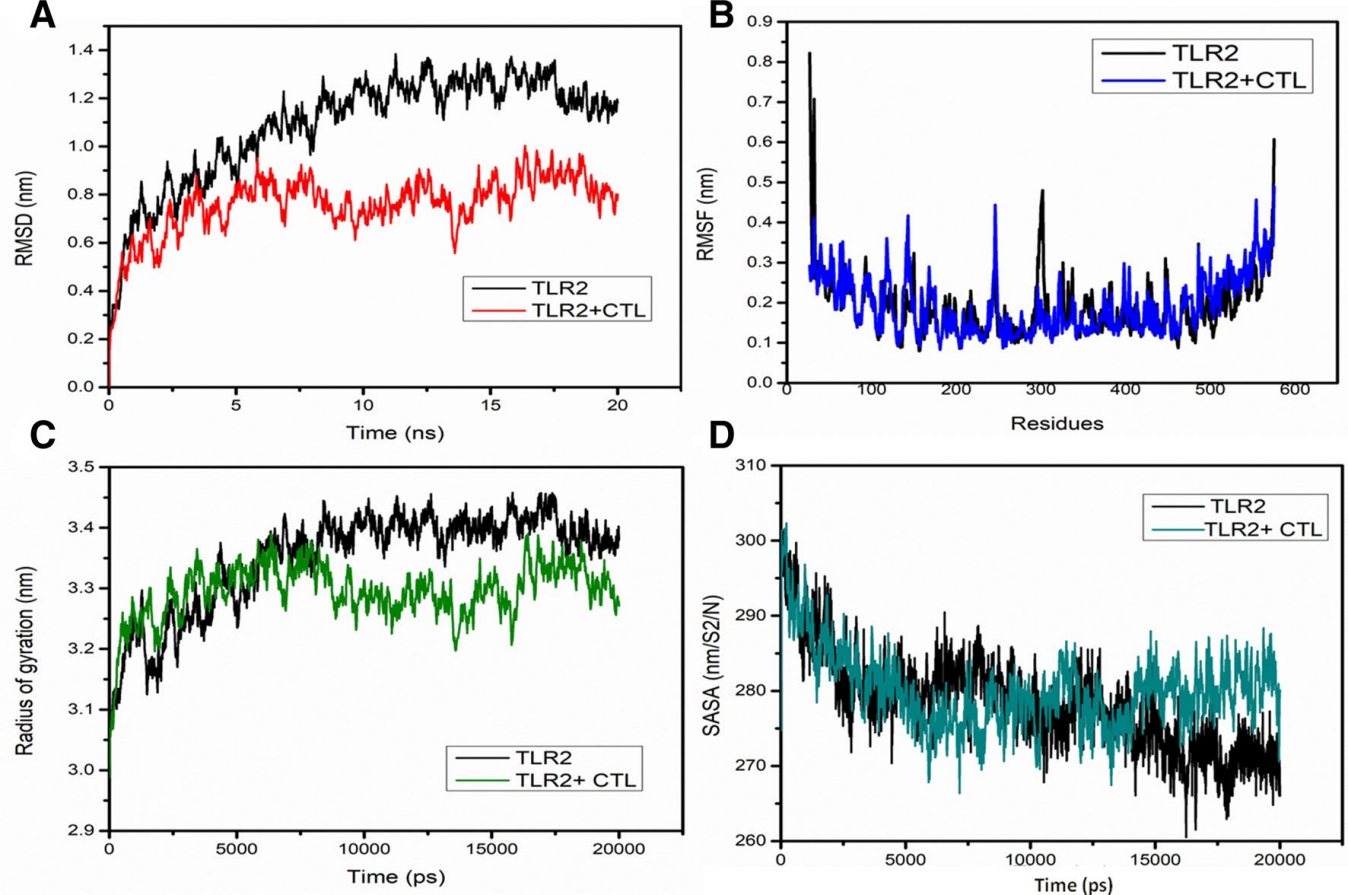
The stability of the TLR2 and TLR2-CTL epitope complex as predicted by molecular dynamics simulations. (**A**) RMSD profile of TLR2 (black traces) and TLR2-CTL(red traces) epitope. (**B**) RMSF profile of TLR2 (black traces) and TLR2-CTL epitope (blue traces). (**C**) Radius of gyration plots of TLR2 (black traces) and TLR2-CTL epitope (green traces). (**D**) SASA profile of TLR2 (black traces) and TLR2-CTL epitope (green traces). The comprehensive computational strategy was utilized to attain insights towards the epitope’s antigenicity against TLR2.

The MD simulations for TLR2 and TLR2-epitope of 20 ns were assessed through trajectory analysis. For the course of 20 ns MD simulation, the stable trajectory was observed and the representative structures were obtained. The deviation of the backbone atoms for simulated structures relative to the starting structures used as a reference was evaluated through RMSD (Fig. 3A). Steep magnitude RMSD variation during the entire simulation can be an implication of a malleable and free instinctive protein or the alteration of the force field. Depending upon the outcomes of the RMSD evaluation, Fig. 3A represents that the RMSD fluctuation leads to stabilize at about 8 ns and 5 ns MD simulations for TLR2 and TLR2-epitope, respectively and the simulation time was acceptable. In the time from 8–20 ns and 5–20 ns, RMSD for TLR2 and TLR2-epitope have approximate values about 1.1–1.3 nm and 0.8–0.9 nm, respectively.

The integral extent of this criterion is achieved by assessing the variations arising from shifts of each of the protein residues that majorly feature the most flexible chain frames. Hence, we validated the residual fluctuations by calculating the mean fluctuation for stable trajectories of each simulation. The RMSF evaluation of all protein residues were achieved in order to check the residues that may have tend to an enhancement in the RMSD results (Fig. 3A). Compelling fluctuations existed in terminal residues and in various loops of the β linker segments of the TLR2 (residues 285-348) to about 0.45 nm and TLR2-epitope (residues 105-156 and 239-254) to about 0.4 nm. Also, we noticed that residues 390 to 480 showed comparatively reduced discrepancy in RMSF values, which were the corresponding binding segment of the TLR2-epitope complex after MD simulation. Interestingly, the binding region for TLR2-epitope before MD simulation was also within the residues 390 to 480, which remains the same after simulation, suggesting as a strong binding interaction of the epitope towards TLR2 and provides stability to the TLR2-epitope (Fig. 3B).

The radius of gyration analysis was performed to determine the change in compactness of TLR2 and TLR2-epitope throughout the MD simulations. The R_g_ plots for TLR2 and TLR2-epitope show slight fluctuations at the initial frame and attains stability after 8 ns and 5 ns with R_g_ score of 3.4 nm and 3.3 nm, respectively which remained approximately similar till 20 ns run (Fig. 3C). When compared with TLR2, R_g_ value for TLR2-epitope is reduced, suggesting more compactness due to the strong binding interaction of the epitope. Similar observations were determined through SASA analysis representing the solvent defined protein surface and its orientation through folding, making the alterations in the exposed and buried regions of the surface area of proteins. SASA values for both the simulation systems were about 280 nm/S^2^/N and 275 nm/S^2^/N after 8 ns and 5 ns, respectively (Fig. 3D). Also, it remains similar till 20 ns in the case of TLR2-epitope, whereas it reduces slightly about to 272 nm/S^2^/N after 15 ns in the case of TLR2. Here, TLR2-epitope solvation profile shows a convincing SASA value suggesting a stable structure and strong binding interaction with the epitope.

**Figure 4:**
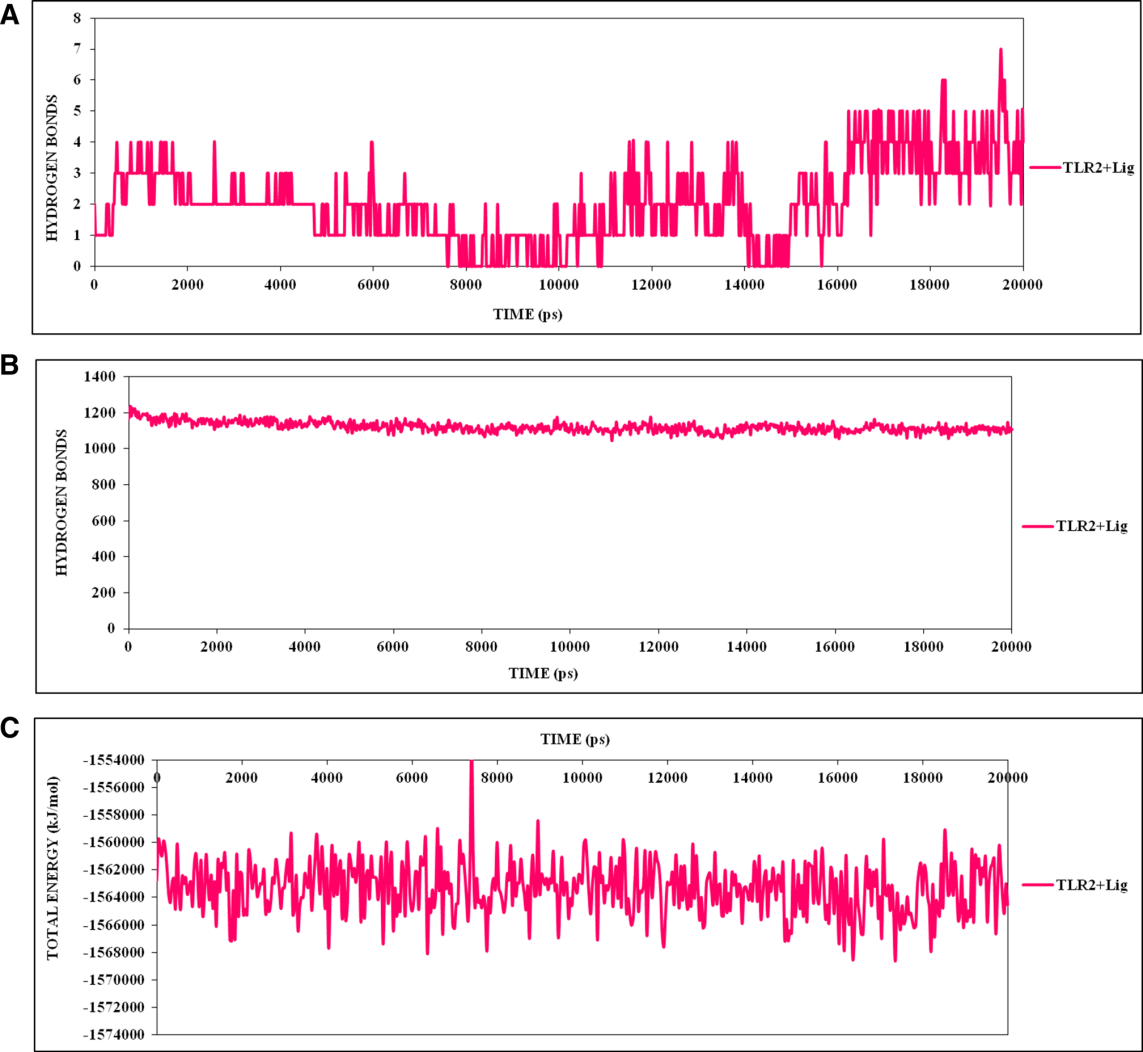
(**A**) The statics of consistent high number of hydrogen bonds involved in strong binding of CTL epitope to TLR2 receptor. (**B**) Depiction of TLR2 receptor-CTL epitope stabilized hydrogen bond throughout the simulation run. (**C**) The binding energies calculation of CTL epitope to TLR2 receptor, which showing the stringent interaction in all cluster frames in 20 ns run.

Further, cluster analysis having a RMSD based cut-off value of 0.25 nm demonstrated the development of five and ten distinctive clusters for TLR2 and TLR2-epitope simulation systems. The most dominant cluster attained after 20 ns of MD simulation for TLR2 and TLR2-epitope are shown in Fig. 4. Also, secondary structure analyses of the stable trajectory for both the simulation systems were performed using the DSSP tool of GROMACS. Both, TLR2 and TLR2-epitope were formed mainly of conserved β-sheet along with various loops of the β linker segments and small coil regions as secondary structure elements infused with various small segments of bend, α-helix, turn, and β-bridge (Fig. 4). Both cluster analysis and secondary structure analysis reveals the conformational changes before and after simulations for TLR2 and TLR2-epitope structures. A noticeable observation through these analyses supports the rationale that protein turns into somewhat unfolded and implements further surface to bind with the epitope.

**Figure 5:**
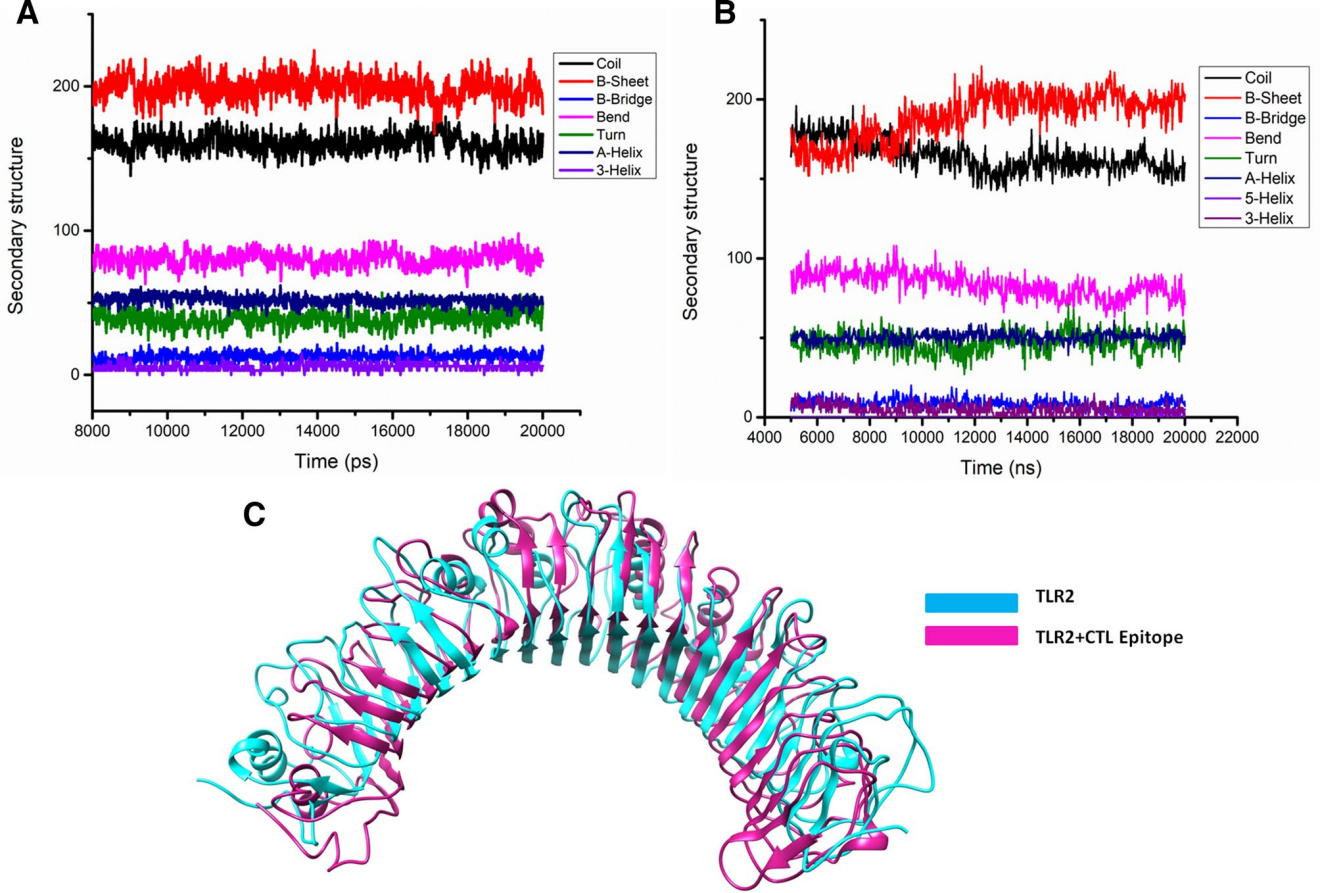
The secondary structure analyses of the stable trajectory for both the simulation systems were performed using DSSP tool of GROMACS (**A**) TLR2, (**B**) TLR2-CTL epitope, (**C**) most dominant cluster of TLR2 (Cyan) and TLR2-CTL epitope (Pink); stable conformation after binding of CTL.

Inaddition, the hydrogen bond landscape was assessed, which revealed the dynamic equilibration of the complex trajectories with a high number of hydrogen bonds, as shown in Fig. 5. The consistent high numbers of hydrogen bonds were observed which contributed significantly to the proximal binding of the CTL epitope with the TLR2 receptor. Further, these results were strengthening by the vital contribution by the complex binding energies throughout the simulation run. These calculations with consistent high binding energies and large hydrogen bonds involvement demonstrated the stable binding of epitope with TLR2.

### In Silico immunization analysis of vaccine construct

**Figure 6:**
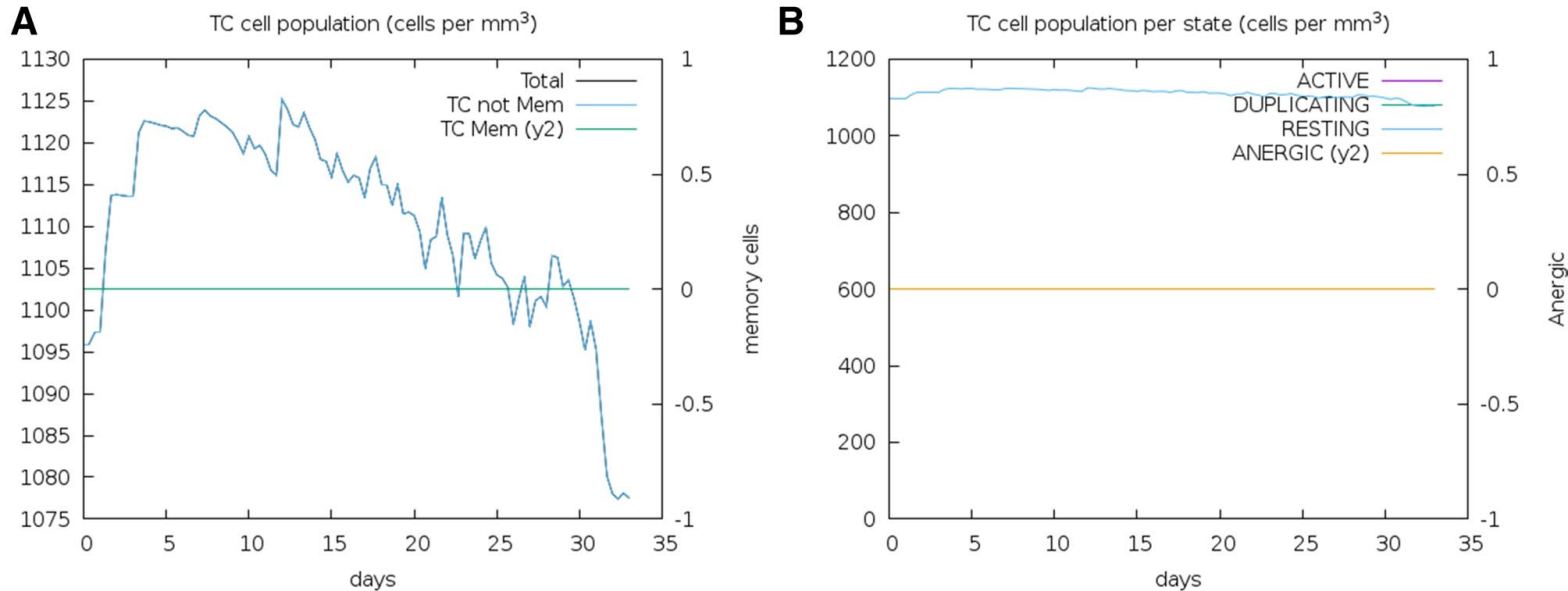
Vaccine constructs in silico immunization analysis. (**A**) CTL response to elicit the Cytotoxic T-cell population for long span. (**B**) Elevated level of Cytotoxic T-cell population in resting state for 30 days.

The vaccine construct was analyzed for immune response capability through the in silico immune simulation approach for 100 simulation steps. The immune simulation method was computed through machine learning with a mesoscopic scale simulator to assess the generation of key regulators of the immune system. Obtained results depicted the substantial elicitation of cytotoxic T-cell (Tc) population in response to vaccine construct. Major regulator immune systems (T-cells) were found to evoke above 1100 cells per mm^3^ and maintained for the long span to 30 days (Fig. 6). Moreover, through the mesoscopic analyses, it was found that T- cytotoxic cells count was perpetuated in resting state (elevated level) till 30 days span and contributed to the memory cells on the immune system, which also prepare the immune system to fight off with the re-infection of specific antigenic determinants. These outcomes demonstrated the potency of immune CTL epitope to be used as a vaccine candidate against COVID-19.

### 3D Structural conformational and physicochemical analysis of lead CTL

**Figure 7:**
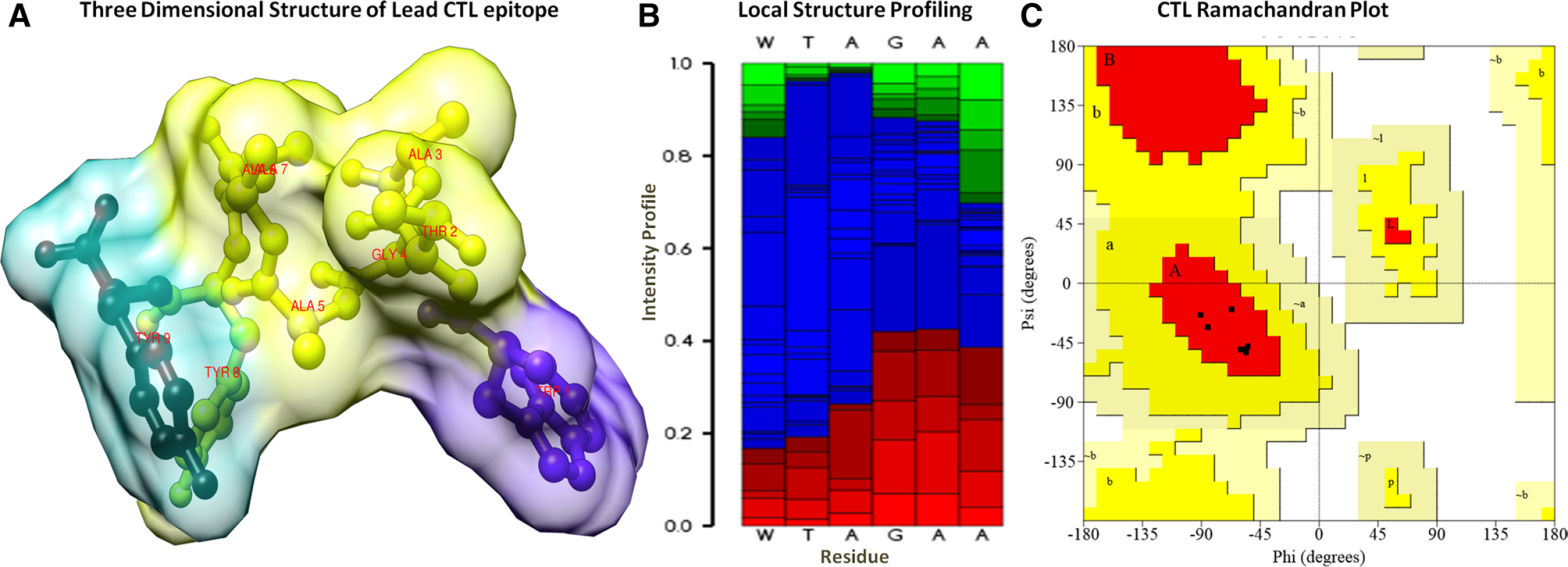
(**A**) The three-dimensional globular structure of CTL epitope (Vaccine candidate). (**B**) Local secondary structure analysis of CTL epitope, Structural profile is shown by helicity (red), extended coils (green) and coils (blue). (**C**) Ramachandran plot analysis of vaccine candidate, depiction residues were lying in a favorable region.

The 3D structure of lead CTL was designed by the Pepfold server and examined through the Ramachandran plot. It showed the existence of structural coordinates in the favorable region of the Ramachandran plot (Fig. 7). The local structure profiling depicted the stable composition of secondary structural coordinates through the probabilities of each residue at each position of the sequence. Moreover, the CTL-5 was found non-allergenic by the Allertop server. The physicochemical properties were investigated by using the Protparam server. The obtained results showed that the lead CTL-5 of size nine amino acids has a molecular weight of 905.03 Daltons and theoretical pH 5.52. The molar extinction coefficient was computed to 28,480 M^−1^ Cm^−1^ with an absorption matrix of 0.1% (1 g/l) 8.715 through the cysteine coupling at 280 nm in solvent condition. Lead CTL-5 was found to be globular nature with an aliphatic index score of 44.44 and classified to stable with an instability index score of − 3.53, the output instability index score less than 40 reported to steady and stable coordinates. Moreover, it was estimated using the computational assays, which showed it has a long span (> 2.8 h) half-life in mammalian reticulocytes by in vitro data statistics, > 2 min for *Escherichia coli* and *Yeast* by in vivo data statics. The GRAVY score (grand average of hydropathy) was computed to be 0.289 and indicated the hydrophilic nature with optimal cellular localization.

## Material and methods

### Coronavirus infectious sequence retrieval and physicochemical analysis

The spike glycoprotein of SARS-CoV-2 is crucial for attachment of it to the host cell and also for arbitrating the infectious propagation of the virus^29^. The spike glycoprotein sequence (with ID GenBank: QHR63290.2) was retrieved from the National Centre for Biotechnology Information (NCBI) (http://www.ncbi.nlm.nih.gov) and assessed for identification of putative cytotoxic T-lymphocytes. Also, the primary sequence alignment of the infectious spike glycoprotein sequence, embracing the conservancy and homology analyses were performed using the BLAST tool^30^.

### Putative cytotoxic T-lymphocyte epitopes determination using five algorithms

CTL epitopes were predicted using five different prediction servers, namely, Rankpep, BIMAS, NetMHC 4.0, NetCTL-1.2, and CTLPred. The distinct algorithms were employed in order to predict more reliable and efficient vaccine candidates. The initial use of the Rankpep server, which is a PSSM based algorithm-generated 32 peptide sequences interacting with the HLA alleles 0201 and having a binding threshold value of 64. Rankpep harbors all the HLA supertypes leading to the generation of potent nanomeric peptide sequences as epitopes. Interestingly, inspection of the results indicated that more than 80% of these predicted epitopes are among the top 2% of scoring peptides^31^. Subsequently, BIMAS was used, which is one of the oldest tools for predicting potential epitopes on the basis of MHC complexation and coefficient ranking of defined epitopes^32^. Following this, the prediction of putative candidates based on their binding affinities was accessed using the NetMHC 4.0 server, incorporating gapped sequence alignments fed to an artificial neural network^33^.

Later on, the NetCTL-1.2 tool was used, which allows the prediction of CTL epitopes restricted to 12 MHC class I supertypes besides integrating the estimation of MHC class I binding to peptides, TAP transport efficiency after the proteasomal C-terminal cleavage. Artificial neural networks were employed in the prediction of MHC class I binding and proteasomal cleavage. TAP transport efficiency was predicted using weight matrix^34^. Finally, CTLPred was used, which allows a direct prediction of CTL epitopes crucial in subunit vaccine design by using the methods based on patterns of T cell epitopes instead of MHC binders. The method broadly involves some elegant machine learning techniques like support vector machine and artificial neural networks^35^. The consensus sequences with a high-rank coefficient in all the predicted epitopes estimated from the aforementioned servers were chosen for obtaining highly authentic and diverse epitopes.

### Selection of lead epitope by immunological parameters

The top-scoring CTLs were examined for the crucial parameters like MHC binding efficacy, C-terminal cleavage efficiency, transporter associated with antigen processing (TAP) transport efficacy, combinatorial rank correlation, antigenicity and toxicity. The netCTLpan server was employed for analyzing the C-terminal cleavage capability and TAP transport efficiency of the shortlisted epitopes. The optimal CTL peptide size range is obtained after the processing of larger peptides/polypeptides by the ubiquitin proteasomal cleavage system. Identified epitopes were further evaluated for their affinity to the TAP protein transporter for efficient ER translocation. A high TAP affinity is a requisite property of an efficient vaccine candidate because TAP proteins help in the transportation of antigenic peptides to the ER where their bonding onto the nascent target MHC I molecule occurs. The netCTLpan server and Tapreg server were employed to analyze the TAP affinity of the predicted epitopes^36^. Later, the shortlisted CTL epitopes were assessed for their immunogenicity and antigenicity profiles by using the Vaxijen V2.0 and Toxin-pred server (http://www.ddg-pharmfac.net/vaxijen/VaxiJen/VaxiJen.html). The Vaxijen server works based on auto cross-covariance transformation for discriminating between non-antigenic and antigenic epitopes^37^. Subsequently, shortlisted epitopes were assessed to determine their binding interaction with the HLA-A*0201 protein and toll-like receptors, mainly TLR2, which is a ubiquitous protein-specific receptor expressed on the host cell surfaces. Also, we have assessed the epitope conservancy and world population coverage.

### Selection of high binder CTL epitopes for HLA-A*0201

The CTLs shortlisted, after analyzing with immunological parameters, were further analyzed for their binding affinity towards the HLA-A*0201 receptor using the automated molecular docking module HPEPDOCK. HPEPDOCK server utilizes a fast rigid-body protein–protein docking based on a hierarchical algorithm, predicting interactions between receptor protein and ligand^38^. HPEPDOCK is constructed upon three techniques namely, rapid Fourier transform correlation, energy conformation clustering and examining the cluster stability by monte carlo simulations. The molecular interactions and other intermolecular associations in the docked epitope-protein complex were determined using Ligplot.

For the execution of the binding analysis, the molecular structure of HLA-A*0201 was retrieved from the protein data bank and was evaluated for its stability using the Ramachandran plot^39^. The HLA-A*0201 structure was conjugated with the MAGE-A4-peptide complex, and hence it was extracted using the Swiss PDB viewer tool. Also, ERRAT server was used for 3D structure verification of HLA-A*0201. The Ramachandran plot was generated using the Rampage server (http://mordred.bioc.cam.ac.uk/~rapper/rampage.php) that helped in determining phi-psi bond distributions of the protein structures under evaluation. The quality of 3D structure and stereochemistry of the protein was then examined using the Prosa web tool by examining the structure's Z-score (the overall quality factor of structure) (https://prosa.services.came.sbg.ac.at/prosa.php)^40^. The quality of protein structure was assessed by Z-score, which helps in comparing the scores of experimentally determined high-quality structures of proteins of the same size. The molecular docking studies were initiated by the preparation of the HLA-A*0201 receptor structure, which comprised the removal of water molecules, unwanted heteroatoms and duplicated chains. Subsequently, the 3D structure of lead epitope was predicted using PEP-FOLD, which is a de-novo 3D structure prediction tool for peptides with sizes ranging from 9 to 36 amino^41^. It foretells the 3D structures on the basis of the Hidden Markov Models, executing a series of simulations starting from the protein sequence that later generates conformations most representative of energy and population.

### Epitope optimization by binding with homing membrane TLR2 receptor

Top HLA-0201 binders were further examined, with host membrane specific TLR2 receptor. TLR2 receptor structure was retrieved from the protein data bank and evaluated for several 3D structural parameters, including the Ramachandran plot. Furthermore, for authenticating the results, molecular binding of the lead epitope with the TLR2 receptor was assessed with multiple molecular docking algorithms using Cluspro and HEX 8.0 servers apart from HEPEPdock.

### Molecular dynamics simulation studies of CTL with TLR2

The toll-like receptor 2 (TLR2) was used separately for two molecular dynamics (MD) simulations, firstly only TLR2 as control and secondly TLR2 in complex with the modeled epitope (TLR2-epitope). Both the MD simulations were implemented through GROMACS v5.0 under the force field GROMOS96 54a7 having water model SPC216 along with the time step of 1 fs for 20 ns^42,43^. The simulation box for each MD simulation were created with having a size of 8.4 × 6.6 × 7.2 nm, which were further loaded with about 43,994 and 43,967 water molecules with SPC model for both TLR2 and TLR2-epitope simulation systems. The total charge on the TLR2 was found to be − 5.000e, therefore to neutralize the negative charge + 5.000e were incorporated into the simulation systems by compensating the water molecules in the arbitrary locations inside the simulation box. NPT ensembles, along with periodic boundary conditions, were utilized to carry out MD simulations. A cut-off of about 12 Å was used in order to manage the Vander Waals forces. The Particle Mesh Ewald model having a cut-off of 14 Å was further utilized to calculate the electrostatic interactions ^44^. TLR2 and TLR2-epitope were solvated through a slab of about 10 Å in every direction. The neighbor list was updated to a frequency of 10 ps.

Each system has gone through four major steps of MD simulations. First step deals with the energy minimization of the entire system utilizing the integrator of steepest descent in continuation with second integrator of conjugate gradients algorithms. Second step involves the minimization and molecular dynamics of NVT and NPT ensembles for 500 ps and 1000 ps, respectively allowing the solvents and ions to evolve and where the starting configuration for the structures were kept same. Third step where the systems were heated having a lower temperature coupling (τ = 0.1 ps) along with pressure coupling (τ = 0.5 ps) to attain equilibrium at 300 K and 1 atm of temperature and pressure. In the equilibration phase, the thermostat and barostat was evaluated through the Berendsen algorithm^45^. The hydrogen-containing bond lengths were constrained with the help of the LINCS algorithm^46^. The last fourth step also called the production step where the MD simulations for 20 ns at 300 K temperature having 2 fs of time step were performed for both systems, and the last final structures were achieved. The Maxwell Boltzmann distribution was utilized in order to reassign the velocities at every step. Nose Hoover thermostat and Parrinello Rahman barostat were the respective thermostat and barostat for the final MD or production run^45^.

Various analyses were performed with the help of inbuilt analysis commands of GROMACS. Root mean square deviation (RMSD) is a magnitude of the dimensional disparity among the two stagnant structures, and RMSD calculation is achieved depending upon the native structure and each consecutive trajectory frames in the simulation. In addition, root mean square fluctuation (RMSF) profile measures the affability of every protein residue depending on the fluctuation about an average location within all MD simulations^47^. Therefore, RMSD and RMSF of both TLR2 and TLR2-epitope complex were determined to examine stability and residual fluctuations. Further, the radius of gyration (R_g_) analysis was performed to evaluate the compactness of both the simulation systems separately. Also, the hydrogen bond analysis was performed to check the neighboring interactions with both simulation systems separately, including the hydrophobic interactions with the help of the LigPlot tool for both TLR2-epitope complexes before and after simulation.

Additionally, solvent accessibility surface area (SASA) was also computed to examine the solvent attributable areas of TLR2 and TLR2-epitope. Cluster analysis having a cut-off value of 0.25 nm depending upon the RMSD profile was utilized to demonstrate the conformations found utmost intermittently throughout the trajectory. Here, all the structures having RMSD values of below 0.25 nm for all components within a cluster are incorporated to the initial cluster. It is rare that a molecule having a higher value for RMSD than 0.25 nm from other cluster supposedly would be treated as a structure. The secondary structure analysis was also performed using the DSSP program^48^. The visualization of protein nature during the entire simulation was accomplished by using Visual Molecular Dynamics (VMD)^49^ and UCSF Chimera^50^.

### In silico immunization analysis of vaccine construct

**S**tudies determining the immune response development by lead vaccine candidate were performed using the in silico immune simulation approach involving 100 simulation steps. Immunization experiments analyzing the immune response generation were based on the combined strategies of system biology and the details obtained by data-driven prediction methods. The C-ImmSim based position-specific scoring matrix, and machine learning approaches illuminated the statistics of antigen response at different time intervals. This approach is potentially based on Miyazawa and Jernigan protein–protein assessment, governing the determination of molecular binding in regard to immune responses^51^. The immune generation capability of the putative vaccine was investigated by simulation of the classical immunization approach and determining the immune system key player cells; cytotoxic T-cells and other memory immune cells.

### Epitope 3D structure optimization and physicochemical analysis

The conformational and structural solidity of epitope structure was estimated by determining the physicochemical properties of the lead CTL epitope. The secondary structural analysis of the designed vaccine candidate was done by Psipred and GOR4 software. The optimization of three-dimensional structure and energy minimization of the putative vaccine was done by the Gromacs minimizer suite. Analysis of the phi-psi angle and stereochemical properties of the epitope were assessed through the Ramachandran plot. The structural and conformational properties of the epitope were investigated by the ProtParam and preLOCFMD servers^52^.

## Conclusion

The conserved spike glycoprotein sequence of the infectious SARS-CoV2 was derived and analyzed using the advanced immunoinformatics approaches. We report the CTL epitope-based vaccine, which has a high pragmatism in vaccine designing, complement the convalescent plasma therapy and can elicit multiple serotype-specific immune responses. The present study concludes that the highly potential CTL epitope vaccine construct has the potential to obtain strong but specific immune responses. The designed CTL epitope was evaluated through the various immunological parameters, reported to comprise in validating the efficacious vaccine. The CTL construct showed the high antigenicity, immunogenicity, and C-terminal proteasomal affinity along with strong TAP affinity. Moreover, the CTL construct showed a strong interaction with the HLA*0201 receptor (MHC-1 restricted) and the host membrane specific TLR2 receptor, which consequently secreted the interferons to destruct the viral proteins.

Interestingly, the molecular interaction of CTL was analyzed through the detailed molecular dynamics studies. Through the trajectory analysis for the long run 20 ns, we examined the RMSD, RMSF, the radius of gyration, SASA, binding energies, and secondary structure contents throughout the simulation run, which suggested the stable binding and compactness due to the strong binding interaction of the epitope with TLR2. Through the in silico immunization, the CTL epitope depicted reliable elicitation of anti-corona immune response. The significant results were found in correlation with optimal physicochemical properties. This study paved the way to a potential CTL based vaccine construct against SARS-CoV-2 through detailed theoretical analysis.

## Data Availability

Data produced and analysed in this study are available from the corresponding author on request.
